# Safety and efficacy of protamine after transcatheter aortic valve replacement

**DOI:** 10.1016/j.ihj.2024.09.001

**Published:** 2024-09-24

**Authors:** Lakshmi Durga Kumaraguruparan, Asuwin Anandaram, Kamalakkannan G. Sambandam, Yogapriya Chidambaram, Bharath Raj Kidambi, Gautam Ganesan Karthikeyan, Madhesh Kasi, Rizwan Suliankatchi Abdulkader, Sankaran Ramesh, Vadivelu Ramalingam, Ravindran Rajendran, Nagendra Boopathy Senguttuvan

**Affiliations:** a Department of Cardiology, Sri Ramachandra Institute of Higher Education and Research (SRIHER), No.1, Ramachandra Nagar, Porur, Chennai, Tamil Nadu, 600116, India; b Department of Internal Medicine, Lehigh Valley Health Network, Allentown, PA, 18101, United States; c Department of Cardiac Anaesthesia, Sri Ramachandra Institute of Higher Education and Research (SRIHER), No.1, Ramachandra Nagar, Porur, Chennai, Tamil Nadu, 600116, India; d Department of Clinical Research, Sri Ramachandra Institute of Higher Education and Research (SRIHER), No.1, Ramachandra Nagar, Porur, Chennai, Tamil Nadu, 600116, India; e Department of Cardiology, AI Dhannah Hospital, Ruwais, Abu Dhabi, United Arab Emirates; f Department of Cardiology, PSRI Hospital, New Delhi, India; g National Institute of Epidemiology, ICMR, Chennai & Department of Statistics, Manonmaniam Sundaranar University, Abishekapatti, Tirunelveli, Tamil Nadu, India; h Department of Cardiology, Velammal Medical College and Hospital, Madurai, India; i Department of Cardiology, Apollo Hospitals, Trichy, India

**Keywords:** Protamine, Transfemoral Trans-catheter aortic valve replacement, Vascular complications

## Abstract

Transfemoral Trans-catheter Aortic Valve Replacement (TF-TAVR) is a safe alternative to surgical aortic valve replacement (SAVR). Protamine is used to reverse heparin and reduce post-TAVR bleeding, but concerns about risks like valve thrombosis and stroke remain. This systematic review and meta-analysis, following PRISMA guidelines, found no statistically significant difference in major bleeding complications between the protamine and control groups [(3.0 % vs. 14.4 %); RR: 0.56; P = 0.16]. No differences were noted in life-threatening bleeding, blood transfusions, 30-day mortality, or stroke. Protamine appears safe post-TAVR without increasing stroke risk, but its effectiveness in reducing bleeding needs further investigation through a multicentric randomized study.

## 1 Introduction

Transcatheter aortic valve replacement is approved for all elderly patients with severe aortic stenosis. Bleeding and vascular complications after TAVR remain significant contributors to mortality and morbidity post-TAVR. Hence, many centers have routinely started using protamine to reverse the effect of heparin post-TAVR. According to the latest data, using protamine sulfate (PS) in TAVR procedures has significantly increased. An analysis conducted by Zbroński et al between March 2010 and November 2016 indicated that 21 % of patients received PS to reverse the effect of unfractionated heparin (UFH) .^1^ In a subsequent study by the same authors, 56 % of patients were administered PS during TAVR procedures.^2^ Our current study, which includes data from two observational studies and one randomized controlled trial, indicates that 65.8 % of patients undergoing TAVR are now administered PS. Additionally, unpublished data from the author's center suggests that the usage of PS has increased to as high as 80 %. This reflects a growing trend towards the routine use of PS in clinical practice to mitigate bleeding risks associated with TAVR procedures.

However, few recent reports suggest the need for caution in using protamine post-valve surgery, citing risks such as stroke, acute valve thrombosis, and anaphylactic reactions.^3^ Only a few small published studies have addressed the question. In this context, we performed a systematic review and meta-analysis to determine the safety of protamine use in patients undergoing TAVR, specifically focusing on its role in reducing bleeding and other vascular outcomes, as well as the associated risk of stroke.

## 2 Methods

The study protocol was registered with PROSPERO, the International Prospective Register of Systematic Reviews (CRD42023417119). Being a meta-analysis of published studies, the study was exempt from the Institutional Review Board. We searched PubMed, Embase, Google Scholar, Clinicaltrials.gov, and the International Clinical Trials Registry Platform (ICTRP). The study's primary outcome was major bleeding, as per the Valve Academic Research Consortium (VARC) definition. According to the VARC, Major bleeding is Overt bleeding that is either associated with a drop in the hemoglobin level of at least 3.0 g/d requiring transfusion of two or three units of whole blood/RBC, or causes hospitalization or permanent injury, or requires surgery and does not meet criteria of life-threatening or disabling bleeding. Whenever data from more than two studies were available, secondary outcomes, including life-threatening bleeding, the requirement for blood transfusions, any stroke in-hospital, stroke or transient ischemic attack (TIA), myocardial infarction, in-hospital mortality, 30-day mortality, vascular thrombotic events, major vascular complications, bail-out balloon strategy usage/covered stent usage were collected. We used The Preferred Reporting Items for Systematic Reviews and Meta-Analyses (PRISMA) statement for the study. Heterogeneity was defined using I^2^. We used the DerSimonian and Laird random effects model for our analysis. All analyses were conducted in R version 4.0.3. A *p*-value of less than 0.05 was considered to be statistically significant.

## 3 Results

**Fig. 1:**
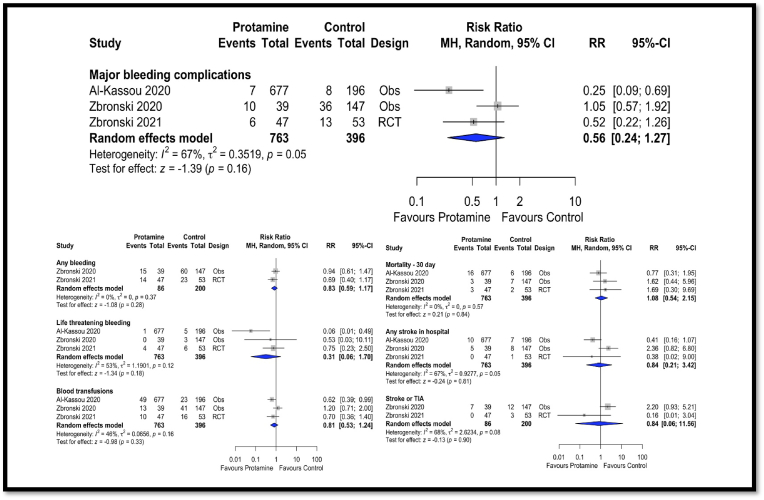
Outcomes with and without protamine post-TAVR.

The final analysis included three studies [2 observational studies and one randomized trial (Zbronski 2021)] with a total patient population of 1159 (Protamine group = 763 & control group = 396).^2^^,^^4^^,^^5^ The primary outcome of major bleeding was numerically more frequent in the control group but was not statistically different from the protamine group. Out of 763 patients in the protamine group, 23 (3.0 %) had major bleeding complications, compared to 57 patients (14.4 %) of 396 control group patients [(3.0 % vs. 14.4 %); RR: 0.56; 95 % CI: 0.24–1.27; P = 0.16, I2 = 67 %; Fig. 1]. Higher heterogeneity was noted in the results. There was no difference between the groups for life-threatening bleeding [(0.6 % vs. 3.5 %); RR: 0.31; 95 % CI: 0.06–1.7; P = 0.18, I2 = 53 %], any bleeding [(33.7 % vs 41.5 %); RR: 0.82; 95 % CI:0.59–1.17; P = 0.28; I2 = 0 %], blood transfusions [(9.4 % vs 20.2 %); RR: 0.81; 95 % CI: 0.53–1.24; P = 0.33, I2 = 46 %], 30-day mortality [(2.9 % vs 3.8 %); RR: 1.08; 95 % CI: 0.54–2.15; P = 0.84, I2 = 0 %], and stroke[(1.9%vs 4.0 %); RR: 0.84; 95 % CI: 0.21–3.42; P = 0.81, I2 = 67 %] and stroke or TIA [(8.1 % vs 7.5 %); RR: 0.84; 95 % CI: 0.06–11.56; P = 0.90, I2 = 68 %](Fig. 1).

## 4 Discussion

In this meta-analysis of three studies, there was no difference between the protamine and the control groups for major bleeding complications, life-threatening bleeding, any bleeding, blood transfusions, 30-day mortality, and any stroke. Several other factors contribute to access site bleeding. The non-modifiable risk factors are age, sex, frailty, diseased and calcified vessels, and size of the vessels, while the modifiable are sheath to artery size, operator experience, ultrasound/fluoroscopy guided access, and managing coagulopathy. Al-Kassou et al observed a reduction in the primary endpoint, a composite of 30-day all-cause mortality and life-threatening and major bleeding, which occurred less frequently in the protamine group (3.2 %) compared with the control group (8.7 %) (p = 0.003).^4^ However, the above study was a single-center, observational study with more patients in the protamine arm, causing a bias. However, they showed no increased MI or stroke with protamine, similar to our study. The RCT in our study showed no statistically significant difference in major bleeding complications between the protamine and control groups. Our result was similar to that of the only randomized study that addressed this question.^2^ The safety of protamine in patients undergoing bypass surgery and carotid endarterectomy gives us additional confidence regarding the usage of the same.^5^ Limitations include fewer studies with small sample sizes, higher heterogeneity, and variations in protamine dosage. Our meta-analysis indicates that using protamine to reverse heparin after Transcatheter Aortic Valve Replacement (TAVR) appears to be safe, with no additional benefit in reducing bleeding complications, particularly major bleeding, nor does it significantly increase the risk of stroke or other major complications. However, the high heterogeneity warrants suitably powered RCT to answer this question dogmatically.

## Funding

None.

## Declaration of competing interest

The authors declare the following financial interests/personal relationships which may be considered as potential competing interests:Dr. Nagendra Boopathy Senguttuvan reports administrative support, statistical analysis, and writing assistance were provided by Sri Ramachandra Institute of Higher Education and Research (Deemed to be University). If there are other authors, they declare that they have no known competing financial interests or personal relationships that could have appeared to influence the work reported in this paper.
